# Normalization strategy for selection of reference genes for RT-qPCR analysis in left ventricles of failing human hearts

**DOI:** 10.1186/s12872-022-02614-9

**Published:** 2022-04-19

**Authors:** Zdenko Červenák, Filip Červenák, Adriana Adamičková, Barbara Šalingová, Andrea Gažová, Ján Kyselovič

**Affiliations:** 1 5Th Department of Internal Medicine, Faculty of Medicine, Comenius University in Bratislava, Ilkovičova 8, 841 04 Bratislava, Slovakia; 2 Department of Genetics, Faculty of Natural Sciences, Comenius University in Bratislava, Ilkovičova 6, 841 04 Bratislava, Slovakia; 3 Institute of Pharmacology and Clinical Pharmacology, Faculty of Medicine, Comenius University in Bratislava, Bratislava, Slovakia

**Keywords:** Failing hearts, Left ventricles, qPCR, Reference gene, Normalization

## Abstract

**Background:**

Quantitative RT-PCR is a valuable tool for assessing the gene expression in different human tissues, particularly due to its exceptional sensitivity, accuracy and reliability. However, the choice of adequate control for normalization is a crucial step, greatly affecting the results of all subsequent analyses. So far, only a few studies were focused on the selection of optimal reference genes in left ventricles of failing human hearts, leading to several disparities in experimental results focused on differential gene expression in this area. Therefore, the main objective of this study was to identify a set of suitable reference genes in normal and failing left ventricle tissues, which could increase the reliability of RT-qPCR-based studies in the future.

**Methods:**

We analyzed the expression of 15 commonly used housekeeping genes (ACTB, B2M, GAPDH, GUSB, HMBS, HPRT1, IPO8, PGK1, POLR2A, PPIA, RPLP0, TBP, TFRC, UBC and YWHAZ) in left ventricles of normal and failed hearts with two-step approach. In the first step, we excluded genes which are variantly expressed using ANOVA-based statistical method. Afterwards, the remaining genes were analyzed using geNorm, NormFinder and BestKeeper algorithms, together with delta Cq method. Finally, the geometric mean of gene rankings across all methods was calculated.

**Results:**

Our analysis identified IPO8 and POLR2A as the most stably expressed genes, whereas ACTB and B2M were found to be expressed variantly, suggesting a potential role of these genes in the pathophysiological processes in failing human hearts.

**Discussion/conclusion:**

Using our two-step approach, we identified and validated two reference genes expressed invariantly in left ventricles of both healthy and failing human hearts, as well as provided a guideline for the selection of reference genes in studies comparing gene expression in these types of tissues.

**Supplementary Information:**

The online version contains supplementary material available at 10.1186/s12872-022-02614-9.

## Introduction

Heart failure (HF) is a serious clinical disorder and the most common reason for hospitalization and death among older adults in Europe and the United States [1, 2]. In numerous types of heart diseases, including ischemic heart disease, dilated cardiomyopathy and coronary artery disease, ventricular remodeling is a major feature of heart failure [3, 4]. As the remodeling progresses, heart changes its shape and morphology, mainly due to pathological processes such as cardiomyocyte hypertrophy, apoptosis, inflammation, and collagen deposition [5, 6]. Although there is a significant progress in understanding of the complex pathophysiology of HF, the biochemical pathways associated with the cardiac remodeling haven’t been fully described yet.

Over the last fifteen years, the differences in gene expression between normal and failing human hearts were thoroughly studied, leading to the identification of many genes potentially involved in the ventricular remodeling [6, 7]. In most such studies, the quantification of mRNA abundance in different heart compartments plays a crucial role and quantitative reverse transcriptase polymerase chain reaction (RT-qPCR) is widely considered the method of choice because of its high sensitivity and reliability [8]. However, this method needs an accurate normalization to differentiate between the technical variations and the true biological differences [9]. Generally, this is achieved by relating the mRNA levels of studied genes to those of internal reference genes (RGs), whose expression is unaffected by the biological event under investigation. Previous studies have shown, that the expression levels of frequently used reference genes, such as GAPDH or 18S rRNA can vary markedly across species, cells or tissues, and are often modulated by the pharmacological treatment and/or experimental conditions [10, 11]. Therefore, using just one gene for the normalization could lead to disparities in results and cause problems in downstream analyses. To avoid this, two or more RGs are commonly used for accurate quantification of gene expression [12].

In order to identify the best-suited reference genes for normalization, several statistical algorithms such as geNorm, NormFinder or BestKeeper were developed and are widely accepted [12–14]. Based on the core properties of these algorithms, they could be divided into (i) pairwise comparison-based (geNorm, BestKeeper, Delta Cq), which compare the expression of genes in pairs regardless of whether the analyzed sample belongs to the control or tested group, and (ii) the variance-based (NormFinder), which statistically evaluate the intragroup and intergroup variation in gene expression. Nowadays, geNorm and NormFinder are the most commonly used and both of them produce reliable results. However, a key assumption that none of the candidate reference genes shows systematic variation in its expression profile across the tested samples, must be fulfilled [15–17]. Since this assumption is very frequently violated in the practice, the candidate reference genes (CRGs) should be tested to confirm that their expression do not vary under the specific experimental conditions [15].

Over the past decade, only a few reports on the identification of stably expressed genes in left ventricles (LV) of failing and control human hearts have been published [18–20]. This is partially due to the restrictive numbers of donors, especially when the healthy heart tissue is concerned. These studies led to the identification of various combinations of RGs used for normalization, depending on differences in the number of samples collected, pathophysiological background of the samples, pharmacological treatment, technology, and normalization strategy. Moreover, the number of CRGs analyzed, together with their metabolic characteristics also vary in different reports [10, 18–23]. Therefore, we believe that the analysis of a larger palette of candidate reference genes in HF samples of LV tissue will provide an important overview of CRGs which should be considered for normalization when analyzing the gene expression by RT-qPCR in LV of failing hearts.

The aim of this study was to identify the suitable genes in HF patient samples with different pathological backgrounds (ischemic heart disease, hypertrophic cardiomyopathy, dilated cardiomyopathy and coronary artery disease) and compare the results with those already published. We analyzed 15 genes commonly used for normalization (ACTB, B2M, GAPDH, GUSB, HMBS, HPRT1, IPO8, PGK1, POLR2A, PPIA, RPLP0, TBP, TFRC, UBC and YWHAZ) and applied a two-step approach, where the CRGs were first tested for their expression variation between the groups with ANOVA and then analyzed by four different algorithms to identify the most stably expressed ones. We also show that the 2 best scoring candidates can be used as reference genes for studying the differential gene expression of MYH6 and MYH7 and discuss the potential role of B2M and ACTB in the pathological processes in LV of failing human hearts.

## Materials and methods

### Biological samples and clinical characteristics

Tissue samples were taken from left ventricular myocardium from non-failing and failing human hearts. Failing hearts were obtained from patients undergoing heart transplantation for end-stage heart failure, resulting from ischemic heart disease (ICM; n = 5, all male), hypertrophic cardiomyopathy (HCM; n = 4, 2 female and 2 male), dilated cardiomyopathy (DCM; n = 10, all male) and coronary artery disease (CAD; n = 9, all male). The clinical characteristics of patients prior to the heart transplantation are summarized in Table 1. Healthy heart tissue was obtained from unmatched donors, (n = 5, 3 female, 2 male) suffering from subarachnoid hemorrhage and head injury. No donor had previous diagnosed heart pathology. The average age of healthy donors was 29 years, (22–44 years). All samples were immediately frozen in liquid nitrogen and store at -80 °C until the RNA isolation.

**Table 1:** Clinical, echocardiographic, and biochemical parameters of patients prior to transplantation

Total number of patients	28	
Sex	Male	25
	Female	3
Age		54.4 ± 8.2
Systolic blood pressure—SBP [mmHg]		112 ± 18
Diastolic blood pressure—DBP [mmHg]		67 ± 11
Heart rate [min^−1^]		79 ± 16
Etiology	Dilated Cardiomyopathy—(DCM)	10
	Coronary Artery Disease—(CAD)	9
	Hypertrophic cardiomyopathy—(HCM)	4
	Ischemic cardiomyopathy—(ICM)	5
Echo	Left ventricular ejection fraction [%]	27 ± 14
	Left ventricular end-diastolic diameter [mm]	68.8 ± 15.8
	Right ventricular end-diastolic diameter [mm]	32.8 ± 5.1
	Interventricular septal end-diastolic diameter [mm]	9.4 ± 2.5
ECG	QRS complex [s]	0.140 ± 0.05
	QT interval [s]	0.42 ± 0.04
Biochemical parameters	NT-proBNP [ng/l]	5551 ± 4259
	Glucose [nmol/l]	7.13 ± 3.4
	Uric acid [µmol/l]	500 ± 192
	Total cholesterol [mmol/l]	4.15 ± 1.08
	Triacylglycerol [mmol/l]	1.56 ± 0.8
	Sodium [mmol/l]	136.3 ± 4.6
	Potassium [mmol/l]	4.4 ± 0.39
	Creatine kinase [µkat/l]	3.54 ± 6.4
	Urea [mmol/l]	9.02 ± 4.3
	Creatinine [µmol/l]	112 ± 35

The data are expressed as mean ± S.D

### Candidate reference genes

For this study, the candidate reference genes were selected based on previous RT-qPCR study [18]. Specifically, ACTB, B2M, GAPDH, GUSB, HMBS, HPRT1, IPO8, PGK1, POLR2, PPIA, RPLP0, TBP, TRFC, UBC, and YWHAZ were analyzed. Detailed information on genes and technology used are presented in Supplementary Table 1.

### RNA preparation and cDNA synthesis

Total RNA from frozen samples was isolated using TriReagent (ThermoFisher Scientific, USA) according to the manufacturer’s instructions. The RNA concentration and purity were evaluated spectrophotometrically using Nanodrop ND-100UV-Vis Spectrophotometer (ThermoFisher Scientific, USA). All samples used in subsequent enzymatic reactions showed a 260/280 nm absorbance ratio of 1.8–2.2. The integrity of the RNA was assessed using Qubit 4 Fluorimeter using Qubit RNA IQ Assay (ThermoFisher Scientific, USA; Additional file 1: Table S2). First strand cDNA was synthesized with High Capacity Reverse Transcription Kit (ThermoFisher Scientific, USA) according to the manufacturer’s manual. For each sample, 200 ng of total RNA was reverse transcribed into cDNA. Reverse transcription reactions were diluted to ~ 1 ng/µl and stored until use at -80 °C.

**Table 2:** Stability values and significance levels of the CRGs

Gene	Variance component		F	Significance	V_B_	V_W_	Stability index	Ranking
	Between groups	Within group						
IPO8	0.002	0.061	0.037	0.810	0.004	0.107	0.00042	1
PGK1	0.013	0.054	0.232	0.538	0.036	0.155	0.006	2
HPRT1	0.104	0.053	1.957	0.358	0.203	0.104	0.021	3
GAPDH	0.134	0.059	2.276	0.089	0.243	0.107	0.026	4
**YWHAZ**	0.623	0.041	15.254	**0.036**	0.775	0.051	0.039	**5**
POLR2A	0.225	0.057	3.928	0.115	0.408	0.104	0.042	6
PPIA	0.275	0.143	1.920	0.255	0.339	0.177	0.060	7
**TBP**	0.244	0.031	7.883	**0.007**	0.695	0.088	0.061	**8**
TRFC	0.448	0.212	2.114	0.374	0.364	0.172	0.063	9
UBC	0.208	0.091	2.283	0.212	0.386	0.169	0.065	10
HMBS	0.735	0.213	3.450	0.147	0.654	0.190	0.124	11
GUSB	0.472	0.121	3.908	0.051	0.757	0.194	0.147	12
**RPLP0**	2.356	0.123	19.167	**0.021**	1.959	0.102	0.200	**13**
**B2M**	2.487	0.093	26.871	**0.0001**	2.499	0.093	0.232	**14**
**ACTB**	3.278	0.114	28.636	**0.007**	2.637	0.092	0.243	**15**

P-values were calculated using Welch’s ANOVA test. In bold, p < 0.05

### Real-time PCR

Real Time PCR reactions were performed using TaqMan Gene Expression Assays (Additional file 1: Table S1) and TaqMan Universal Master Mix II (both ThermoFisher Scientific, USA). Each reaction contains ~ 1 ng of cDNA, 0.5 × TaqMan Gene expression Assay, and 1 × TaqMan Universal Master Mix II in 20 µl total volume. Reactions were amplified on QuantStudio™ 5 Real-Time PCR System (ThermoFisher Scientific, USA) in triplicate for each sample and PCR cycling conditions were used according to manufacturer’s instructions. The amplification efficiency (Additional file 1: Table S1) was assessed using LinRegPCR software [24]. The adjusted amplification efficiencies were then used for all calculations in QBASE+ software. To control for the presence of residual DNA, the 200 ng of total RNA was treated with RNAse A (Qiagen GmbH, Germany), reverse transcribed and qPCR reactions were performed as described above. No amplification was observed, indicating no genomic DNA contaminations in the RNA samples.

### Reference gene stability evaluation

The expression of 15 selected CRGs was tested as proposed in [15]. For each CRG, the Cq values were converted to relative expression ratios (Rs), followed by transformation to their natural logs, and subjected to one-way ANOVA. The mean squares of groups (MSB) which represent the intergroup variance caused by differences between groups and mean square errors (MSW), which represent the intragroup variance, were calculated. Consequently, the MSB and MSW of each CRG were divided by -1/X̄ to calculate the intergroup variation index (VB) and intragroup variation index (VW) for each CRG (X̄ is the average of natural logarithmic transformed relative expression ratios of the corresponding CRG). A stability index was subsequently computed by multiplying VB by VW. Afterwards, all CRGs with p-value levels below 0.05 were excluded from further analysis, while the remaining CRGs were analyzed employing four different approaches:

1. We ranked the CRGs by their M-value (average pair-wise variation of specific gene with all of the other analyzed genes) calculated using geNorm algorithm (implemented as a module in Qbase software).
2. We used NormFinder algorithm (included as add-in excel sheet) to calculate the stability values of CRGs and rank them accordingly (the lowest stability value represents the most stably expressed gene).
3. The BestKeeper software was used to calculate Pearson coefficient and p-value for each CRG (for each gene, p-value was < 0.05). Subsequently, Pearson coefficients were correlated with BestKeeper index and the resulting correlation coefficients were used as the basis for the ranking (the highest correlation coefficient was scored as no. 1 in the ranking list).
4. Delta Cq method was used to calculate mean ΔCq and SD across all pairwise CRG comparisons. Subsequently, mean SD was calculated across all comparisons of the gene to all of the other remaining CRGs and the gene with the lowest mean SD was considered the most stable one.

The final ranking of CRGs was then calculated as a geometric mean of ranking of each individual CRG in all particular rankings and the top two CRGs were considered the most stable genes in our dataset (Additional file 1: Supplementary Fig. 1) [18].

### Statistical analysis

Stability indexes in ANOVA frame were calculated using XLSTAT software [25] on amplification efficiency-corrected and log transformed data as proposed in [15]. As the samples in the groups were disbalanced, the p-values of individual CRGs were assessed with Welch’s ANOVA test. Raw amplification data (Cq values) from qPCR were exported into QBASE+ software [26], normalized against geometric mean of the two most stable genes, log transformed and the Mann–Whitney U test was performed to evaluate statistical differences in two independent groups using QBASE+ statistical module. The power of the test was assessed by XLSTAT software. The p-values ˂0.05 were considered as significant. The numerical data were visualized with GraphPad software [27] as mean ± S.E.M.

## Results

The expression ranges of all CRGs in 33 samples from left ventricles of human hearts (5 healthy, 28 diseased) are shown in Additional file 1: Table S3 (for more details see also Additional file 1: Tables S4 and S5). All selected genes are expressed in human myocardium and their respective Cq values vary significantly with B2M and GAPDH exhibiting the highest expression levels and GUSB showing the lowest expression. The smallest Cq range was associated with HPRT1 gene, whereas ACTB showed the largest. The stability of CRGs was first analyzed using ANOVA-based approach (Table 2), showing a variability of stability indexes, ranging from 0.00042 (IPO8) to 0.243 (ACTB). Importantly, this analysis also revealed a significant difference in gene expression of B2M, YWHAZ, TBP, RPLP0 and ACTB between the tested groups, questioning their reliability as RGs. Based on these data, the five genes were excluded from further analyses.

**Table 3:** Final rankings of the candidate reference genes

Final ranking	Distribution of diseased samples		
	Healthy vs. failing	Random groups of 5	Individual cardiomyopathies
**1**	**IPO8**	**IPO8**	**IPO8**
**2**	**POLR2A**	**POLR2A**	**POLR2A**
3	PGK1	UBC	GAPDH
4	UBC	GAPDH	HPRT1
5	HPRT1	PGK1	UBC
6	GAPDH	PPIA	PGK1
7	PPIA	HPRT1	PPIA
8	GUSB	TBP	GUSB
9	HMBS	HMBS	HMBS
10	TFRC	GUSB	TRFC
11	-	TRFC	-

The final raking was calculated as a geometric mean of ranking positions from (ANOVA, geNorm, NormFinder, BestKeeper and Delta Cq method), the two most stably expressed genes are shown in bold

**Fig. 1:**
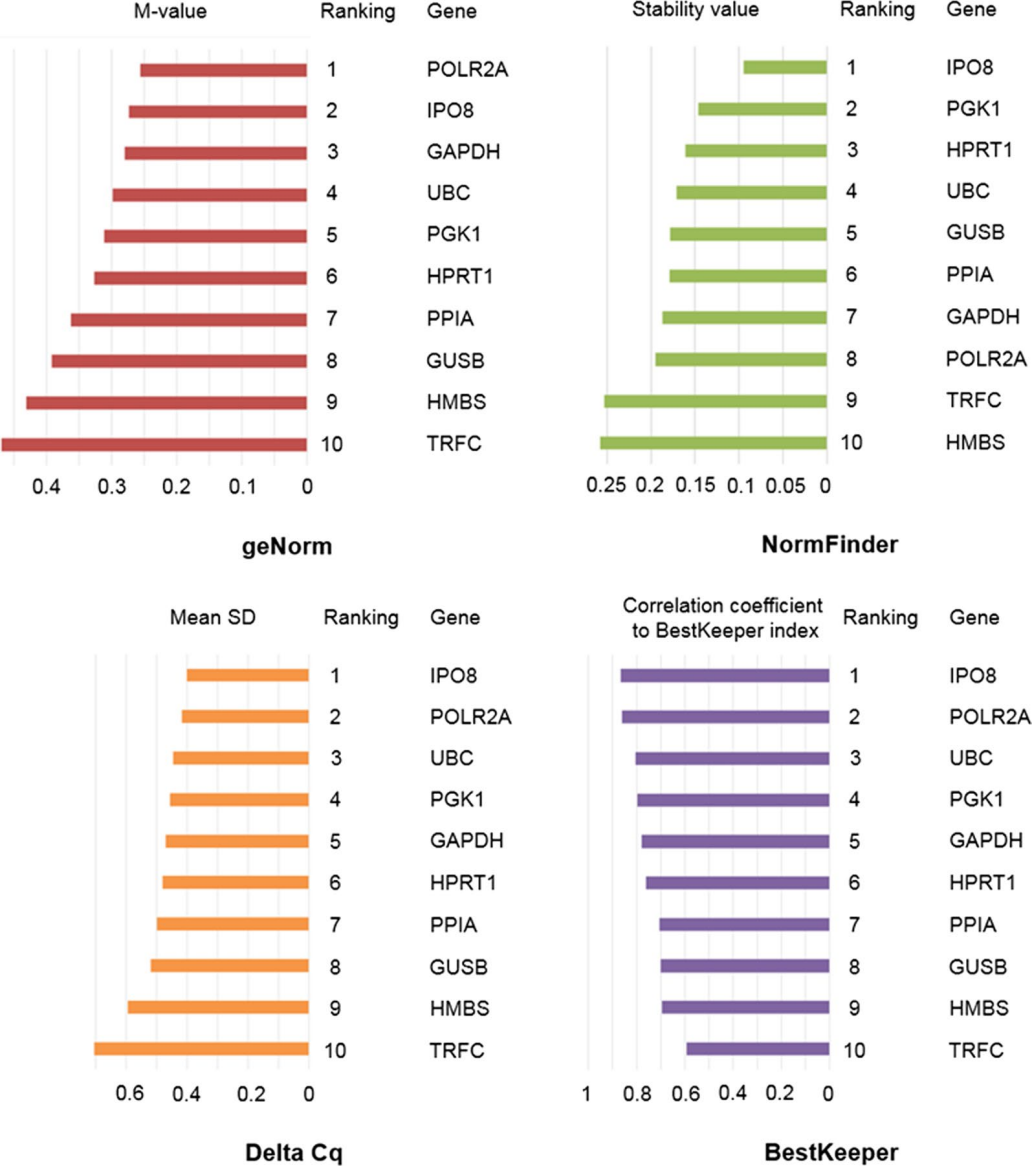
Individual ranking lists based on the geNorm, NormFinder, Delta Cq and BestKeeper analyses of CRGs expression. The coloured bars represent the indexes of stability (M-value, Stability value, Mean SD, Correlation coefficient to BestKeeper index, respectively) calculated in each analysis. The ranking position of each gene is indicated

Remaining 10 CRGs were analyzed with geNorm, NormFinder and BestKeeper algorithms, as well as the Delta Cq method. When comparing the pairwise comparison-based systems (geNorm, BestKeeper and Delta Cq), it is clear that all of them scored IPO8 and POLR2A among the most stably expressed genes (Fig. 1, Additional file 1: Table S6). Moreover, when analyzed with geNorm, all selected CRGs showed M-values below the threshold of 0.5, indicating high stability (Additional file 1: Table S6). On the other hand, the ranking of several genes such as GAPDH or HMBS remains questionable since their position varies between the individual ranking lists (Fig. 1). As a complementary approach, we employed the variance-based NormFinder algorithm, which accounts for variation in gene expression among and within the sample groups and avoids the misinterpretation of data caused by artificial selection of co-regulated genes [13]. Interestingly, the comparison of stability values generated by NormFinder suggests the combination of GUSB and UBC as the optimal set of RGs, since they showed the lowest combined stability value. Individually, IPO8, PGK1 and HPRT1 were listed as the three most stably expressed CRGs (Fig. 1). Taking to account the position in every scoring list, we calculated the final ranking for each tested CRG (using the geometric mean). As expected, IPO8 was identified as the most stably expressed gene in the entire dataset (each method except for geNorm identified IPO8 as the most stably expressed gene). The positions of other tested CRGs were much more variable, although multiple algorithms proposed POLR2A (geNorm, BestKeeper, Delta Cq) as the other CRG with high stability of expression (Fig. 1, Additional file 1: Table S6).

In order to compensate for the disparity in sizes of the analyzed groups of samples (5 healthy vs. 28 failing), we performed another analysis, randomly distributing the diseased samples into 5 groups of 5 samples and comparing the variance in the transcription levels of individual genes between all 6 groups (5 failing and control) using F-ANOVA (Additional file 1: Table S7). Using the resulting data in the calculations, the final ranking showed the same two genes (IPO8, POLR2A) at the top of the list and UBC (originally scored fourth) at the third position (Table 3). Furthermore, we also redistributed the diseased samples into 4 groups based on the specific types of cardiomyopathies (HCM, ICM, DCM, CAD) and analyzed these groups with Welch’s ANOVA (Additional file 1: Table S8). In concert with all of our previous analyzes, the final ranking showed IPO8 and POLR2A as the most stably expressed genes, underlying their status as the top two CRGs (Table 3).

Taken together, our combined approach, employing a variety of algorithms and tests to compare the stability of putative CRGs showed differences in expression levels of some genes between healthy and diseased human hearts and propose IPO8 and POLR2A as the optimal RGs for RT-qPCR studies of mRNA levels in left ventricles of failing human hearts.

### Analysis of MYH6 and MYH7 genes expression using top two CRGs

**Fig. 2:**
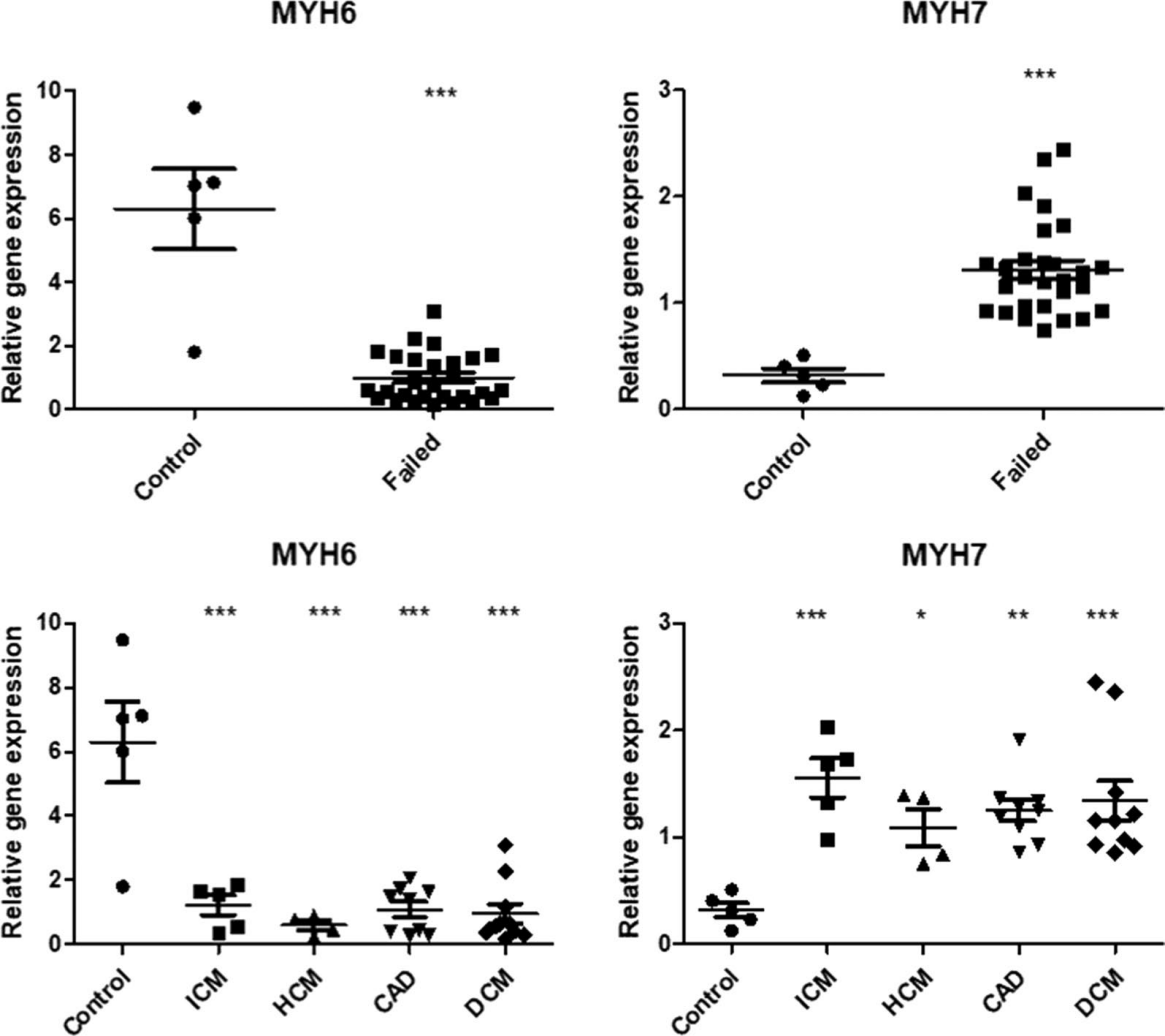
Relative expression of MYH6 and MYH7 genes in LV of healthy and failing human hearts. In the upper panels, the control samples are compared with all of the diseased samples, in the lower panels, the individual pathologies are analyzed separately (HCM—hypertrophic cardiomyopathy, DCM—dilated cardiomyopathy, ICM—ischemic cardiomyopathy, CAD—coronary artery disease). Plots represent the mean ± SEM gene expression ratio of both genes normalized to IPO8 and POLR2A. Data were statistically analyzed using Mann–Whitney test (upper panels) or ANOVA with Dunnett’s post hoc test (lower panels). * indicates p ≤ 0.05, ** indicate p ≤ 0.01, *** indicate p ≤ 0.001

To validate the top-scoring CRGs, we tested our normalization strategy in RT-qPCR analysis of MYH6 and MYH7 genes expression in LV of failing human hearts. MYH6 and MYH7 genes encode the two myosin heavy chain isoforms (α-MHC and β-MHC, respectively), whose expression is altered in diseased tissues and therefore represent a suitable control for our experimental strategy. Specifically, MYH6 gene is significantly downregulated in failing heart tissue, while MYH7 is upregulated [28–30]. As expected, the results presented in Fig. 2 and Additional file 1: Table S9 showed that the transcription of MYH6 gene is downregulated on average 7,5x, while MYH7 is upregulated 4.5x, which is in agreement with previously published data [28–30] and further support IPO8 and POLR2A as reliable RGs. The power of the test reached 100% for both genes. Furthermore, we compared our data with two previously published RNAseq datasets (GSE55396 [31] and GSE116250 [32]) and found that in both of them, MYH6 is downregulated in failing hearts, which is in line with our data, although the exact fold change slightly differs (Additional file 1: Table S10).

### Analysis of ACTB and B2M genes expression using top two CRGs

**Fig. 3:**
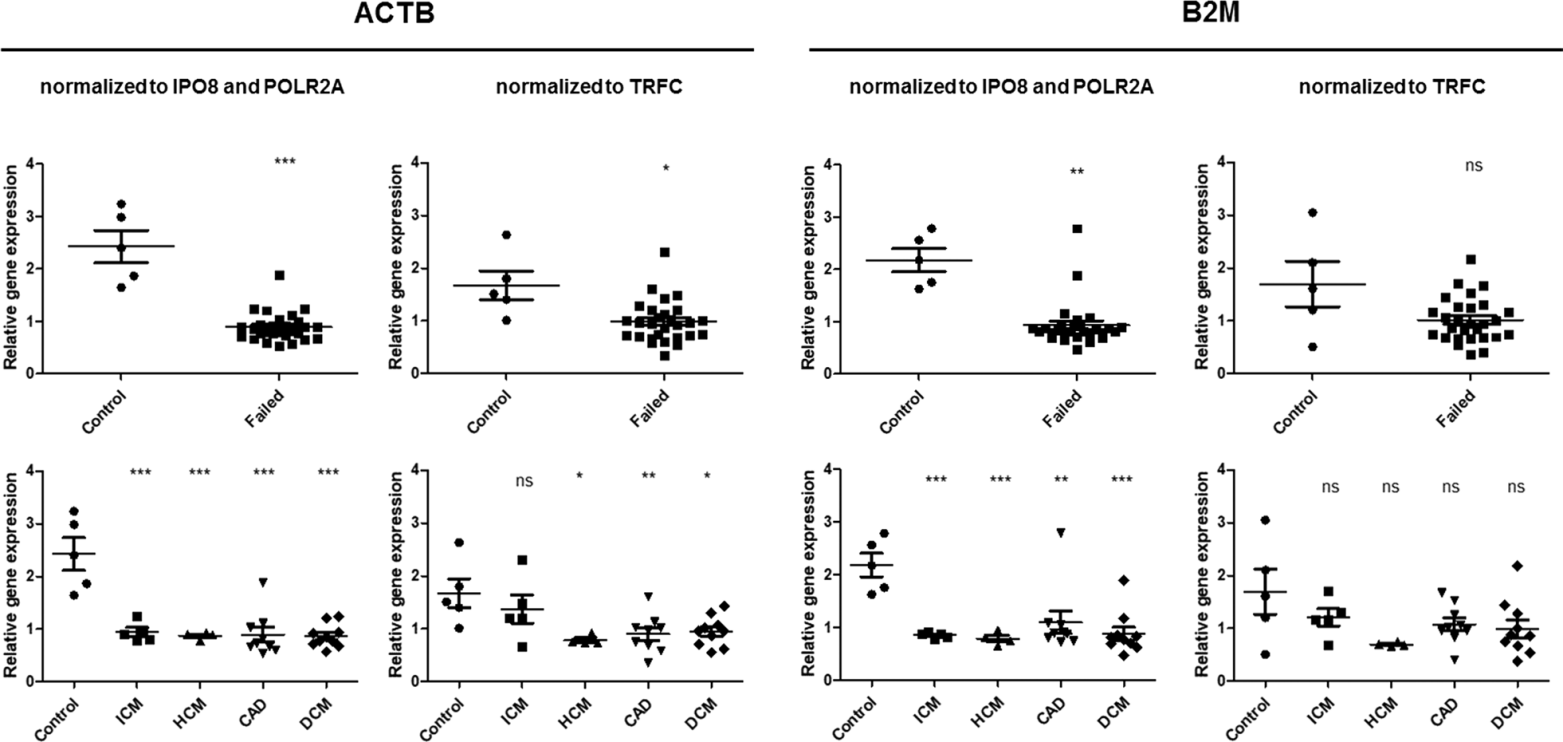
Relative expression of ACTB and B2M genes in LV of healthy and failing human hearts. In the upper panels, the control samples are compared with all of the diseased samples, in the lower panels, the individual pathologies are analyzed separately (HCM—hypertrophic cardiomyopathy, DCM—dilated cardiomyopathy, ICM—ischemic cardiomyopathy, CAD—coronary artery disease). Plots represent the mean ± SEM gene expression ratio of both genes normalized to the indicated RGs. Data were statistically analyzed using Mann–Whitney test (upper panels) or ANOVA with Dunnett’s post hoc test (lower panels). * indicates p ≤ 0.05, ** indicate p ≤ 0.01, *** indicate p ≤ 0.001, ns indicates non-significant differences between groups

The analyses of CRGs in several previous reports showed that ACTB and B2M genes, often used as RGs, are among the least stably expressed CRGs in human left ventricles, regardless of clinical or experimental conditions used [18, 20, 21]. This is in concert with our data, which showed significant differences in expression of these genes in LV of healthy and failed heart tissue (Table 2). Furthermore, we compared the levels of mRNA of both genes by RT-qPCR, using IPO8 and POLR2A as RGs (Fig. 3, Additional file 1: Table S11), revealing a significant down-regulation of both genes in left ventricles of failing hearts regardless of the specific pathology (the power of the test for both genes reached 100%). This observation suggest that B2M and ACTB are not suitable as RGs in gene expression studies aimed at LV of failing human hearts and the changes in their expression might be connected to pathological processes leading to heart failure. Furthermore, when comparing the expression of both genes normalized to IPO8 and POLR2A to the same data normalized to TRFC (one of the less stable genes), it is clear that the two best ranked CRGs allow much clearer clustering of individual samples and reduced variability in each individual sample group (Fig. 3).

## Discussion

The RT-qPCR is a powerful tool to study gene expression at mRNA level. As many factors can influence the accuracy of results obtained by this method (RNA quality, efficiency of reverse transcription, primer design), a correct normalization of primary data is necessary to reduce the variations caused by experimental and analytical procedures. Accordingly, the selection of proper reference genes is crucial, as these genes directly influence the interpretation of RT-qPCR results. Furthermore, in clinical HF samples, the variations in gene expression can be also affected by the number of samples analyzed, specific pathophysiology of the samples and pharmacological treatment, as well as the normalization strategy. Therefore, it is not surprising that the final choice of RGs selected for normalization in various studies of LV from normal and failing heart tissues differ significantly [10, 18–23, 33, 34].

In this study, we selected 15 most commonly used CRGs and compared their stability in both the healthy and diseased samples. First, we employed an ANOVA-based approach [15] to calculate the stability index for each CRG (Table 2). Based on these calculations, IPO8, PGK1 and HPRT1 were considered the most stable genes, with nonsignificant p-values and the lowest intergroup variance. This analysis also revealed that 5 genes (TBP, YWHAZ, ACTB, B2M, RPLP0) were expressed variantly (p ˂0.05) and thus were excluded from further analyses. Among these genes, TBP was previously reported as stably expressed only in post mortem human cardiac muscle tissue [21], while in other studies where it was analyzed, it was never ranked among the most stably expressed genes [18, 33]. On the other hand, YWHAZ was a top–ranked RG when samples from left and right ventricles of patients with LVAD (left ventricular assist device) or HT (heart transplantation without previously implanted LVAD) were tested [22]. Moreover, along with GAPDH, IPO8, POLR2A and PPIA, YWHAZ was identified as a suitable RG in several heart cavities and disease conditions [18]. In contrast, the identification of endogenous controls in LV of hearts from organ donors ranked YWHAZ among the less stable genes [33], which is in concert with our analysis and questions its role as RG in studies aimed at the differential gene expression in LV of failing hearts. The RPLP0 gene was described as suitable RG in study of hearts from human donors [32], but not in the study concerning normal and diseased hearts [18]. Regarding these genes, however, we would like to note that no data have been reported as to whether they had been tested for systematic variation or not. Therefore, we cannot exclude the possibility that in other clinical studies on LV of failing human hearts including different sets of cardiomyopathies and/or different sizes of both control and diseased groups, no statistically significant differences between the tested groups will be observed. Importantly, this does not apply to the last two genes, ACTB and B2M (see below).

Afterwards, the 10 stably expressed CRGs were further analyzed with geNorm, NormFinder and BestKeeper algorithms, as well as the Delta Cq method. Since geNorm, NormFinder or a combination of both is used in the majority of studies searching for suitable RGs, we used them as the primary tools and compared the resulting ranking lists with those generated by BestKeeper and Delta Cq (Fig. 1). Both of these algorithms provide reliable results if none of the evaluated genes shows a systematic variation in the expression profile during the experiment. Unfortunately, there is no way to predict whether a particular CRG is expressed invariantly before performing the experiment and the preliminary analyses of CRGs are frequently based on publicly unavailable microarray or RNAseq data. One way to overcome this problem is to use the statistical approach, which evaluates each CRG individually and identifies genes with systematic variation in gene expression. Using all 5 aforementioned methods, we generated individual ranking lists for each algorithm and eventually combined them into the final ranking. This analysis identified IPO8, and POLR2A as the most stably expressed genes, suggesting they might serve as reliable RGs in RT-qPCR analyses (Table 3).

Among the other seven genes (ranked 4.-10.), HPRT1 has been previously selected for normalization of gene expression profiles in cardiac tissue and blood cells in the study of myocarditis [10, 19] and PPIA was shown to be a functional internal control in study of healthy organ donors [33] and cytokine expression in patients with LVAD [22]. Interestingly, the results on GAPDH are contradictory, as it was reported as a reliable RG in three separate studies [19, 20, 33], while in one report it was found to be the least stable gene [34]. In our study, GAPDH was scored as a slightly less stable gene, ranked sixth in the final calculation. This is in agreement with the results of [18], where GAPDH was ranked as a less stable gene in LV of control and failing hearts. On the other hand, the same study showed that GAPDH is a stable reference gene across different heart cavities and disease conditions [18]. GUSB has been analyzed only in one study, where it was described as the second most stable gene in LV of human controls and failing heart tissues [18]. This is inconsistent with the results of our analysis, where GUSB was ranked among the less stable genes. The UBC gene was analyzed in three studies [18, 21, 33], always found as a less stable, whereas the HMBS gene was reported as a suitable RG in postmortem human cardiac muscle tissue, but not in sudden cardiac death (SDC) analysis [21, 23]. Together with TFRC gene, HMBS was found as the least stable CRG in our analysis, occupying the last two positions of the final ranking.

Finally, when comparing the top three RGs from our study (IPO8, POLR2A and PGK1) with previously published reports, IPO8 and POLR2A were described as suitable RGs in left ventricles of failing hearts and also in all heart chambers and disease conditions [18]. PGK1 has been also reported as appropriate internal control in two analyses of human heart failure [10, 18], suggesting all of these genes might serve as reliable RGs. However, based on all three final ranking lists, including additional ANOVA analyses, we propose the combination of IPO8 and POLR2A as the most reliable set of RGs.

### Analysis of MYH6 and MYH7 genes expression using IPO8 and POLR2A as RGs

To experimentally test this combination of RGs, we analyzed the expression of MYH6 (downregulated in LV of failing hearts) and MYH7 (upregulated in LV of failing hearts) genes by RT-qPCR. Even though the control samples showed a slight variation in expression of MYH6, the expression of both MYH6 and MYH7 was significantly altered in LV of failing hearts, showing the expected patterns of differential regulation (Fig. 2). We also compared our data with two publicly available RNA seq datasets, showing similar differences in gene expression (Additional file 1: Table S10). These data indicate that selected RGs are appropriate for examining the differential gene expression in proposed experimental set up and might be used in RT-qPCR analyses aimed at the identification of both upregulated and downregulated genes of interest.

### Analysis of ACTB and B2M genes expression using IPO8 and POLR2A as RGs

ACTB and B2M genes are very frequently used as RGs in gene expression studies. In our analysis, however, both showed a highly variant expression and were excluded from the dataset in the first step due to low p-values and high ANOVA stability indexes (Table 2). Similarly, these genes were also found as less stably expressed in other studies focusing on the identification of RGs in LV of failing human hearts, regardless of their clinical background [10, 18, 20, 21, 33]. Interestingly, the significant decrease in expression of these two genes might be directly associated with the pathophysiology of the LV of failing heart tissue.

In case of B2M gene, it has been recently shown that high levels of B2M protein in serum are associated with severity of coronary artery disease without renal dysfunction [35]. In addition, B2M levels are higher in plasma of patients hospitalized with chronic heart failure and correlate with the severity of cardiorenal failure [36]. Recently, the increased expression of B2M in stressed mouse cardiomyocytes was reported to stimulate a wound healing response in mouse fibroblasts with induced ischemia–reperfusion injury [37]. In contrast to these observations, we found the B2M mRNA levels decreased in failed hearts. One possible explanation of this discrepancy is that decreased expression of B2M gene could lead to a decreased ability of wound healing in the end-stage human hearts. However, the exact role of B2M gene in pathophysiology of human hearts remain to be investigated.

The role of ACTB gene in failing human hearts is also not yet fully understood. ACTB is one of the cytoskeletal proteins and participates in many important cellular processes, including muscle contraction, cell motility and division, organelle movement, the establishment and maintenance of cell junctions and cell shape [38]. The disorganization of cytoskeletal proteins and loss of sarcomeric structures contribute to the pathogenesis of contractile dysfunction in mammalian hearts and the decreased expression of this gene might lead to the loss of contractile force in end-stage hearts and subsequent heart failure [39]. Moreover, in many eukaryotic cells, β-actin (protein product of ACTB gene) is also present in nucleus, where it regulates the expression of specific genes and processes connected to cell division, and proliferation [40, 41]. One of the best-characterized examples of β-actin role in the regulation of nuclear complexes is its involvement in regulation of MRTF (myocardin-related transcription factor) system, an activator of SRF (serum response factor). SRF is a regulator of cardiac gene expression in response to hypertrophic stimuli and several processes which reduces the monomer-actin (G-actin) lead to the retention of MRTF in nucleus and SRF activation. This in turn could lead to the expression of genes responsible for cardiac remodeling [42, 43]. Interestingly, the cardiac remodeling might be also regulated via interaction of β-actin with endothelial nitric oxide synthase (eNOS). The association of eNOS with β-actin increases eNOS activity and the nitric oxide (NO) production [43]. The following activation of cGMP-dependent protein kinase (PKG) by NO might inhibit the hypertrophic signalling pathways and act as a pro-hypertrophic brake [5, 44].

## Conclusion

In this study, we identified the most stably expressed reference genes for normalization of transcription profiles in LV of healthy and failing human hearts of different pathological background. We analyzed 15 commonly used housekeeping genes and using our two-step approach we identified IPO8 and POLR2A as the most stably expressed CRGs, suggesting them as the optimal reference genes for future studies. We also confirmed that ACTB and B2M are expressed variantly and therefore should not be used as RGs.

## Supplementary Information

**Additional file 1.** RT-qPCR analysis and ranking of CRGs.

## Data Availability

All data analyzed in this study are included in the article and/or its supplementary materials. Further enquiries can be directed to the corresponding author.
