# Supplementary material for: Normalization strategy for selection of reference genes for RT-qPCR analysis in left ventricles of failing human hearts
Source: BMC Cardiovasc Disord. 2022 Apr 19;22:180. doi: 10.1186/s12872-022-02614-9 (PMC9019989; doi:10.1186/s12872-022-02614-9)
Supplement: Supplementary file 1 — Additional file 1. RT-qPCR analysis and ranking of CRGs. [file 12872_2022_2614_MOESM1_ESM.docx]

***Supplementary Material***

***Normalization strategy for selection of reference genes for RT-qPCR analysis in left ventricles of failing human hearts***

Zdenko Červenák^a^, Filip Červenák^b^, Adriana Adamičková^a^, Barbara Šalingová^a^, Andrea Gažová^c^, Ján Kyselovič^a^

^a^ 5^th^ Department of Internal Medicine, Faculty of Medicine, Comenius University Bratislava, Slovakia

^b^ Department of Genetics, Faculty of Natural Sciences, Comenius University in Bratislava, Bratislava, Slovakia

^c^ Institute of Pharmacology and Clinical Pharmacology, Faculty of Medicine, Comenius University Bratislava, Slovakia

**Supplementary Figure 1**

Pipeline depicting the process of identification of the optimal RGs.

**
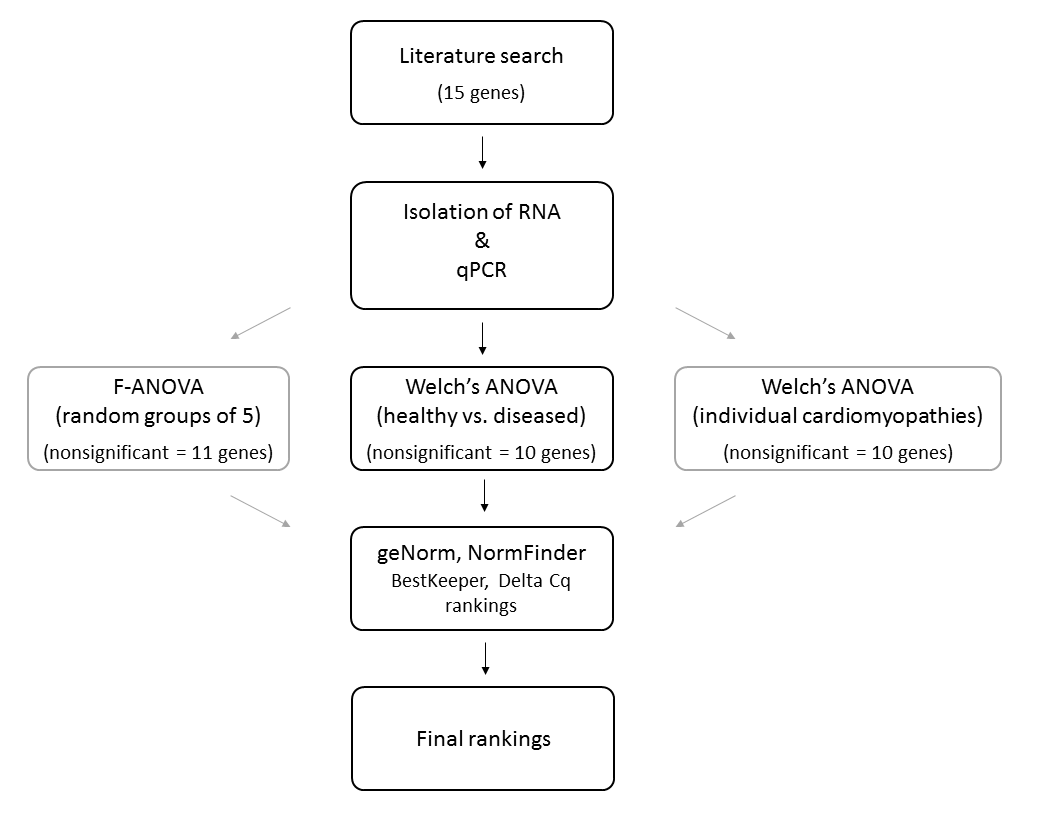
**

**Supplementary Table 1**

TaqMan Gene Expression Assays used for RT-qPCR amplification, amplicon lengths and the corresponding amplification efficiencies.

| **Gene** | **Gene name** | **TaqMan GE Assay ID** | **Amplicon length** | **Amplification efficiency** |
| --- | --- | --- | --- | --- |
| **ACTB** | β-actin | Hs99999903_m1 | 171 | 1.72 |
| **B2M** | β-2-microglobulin | Hs99999907_m1 | 75 | 1.839 |
| **GAPDH** | glyceraldehyde-phosphate dehydrogenase | Hs99999905_m1 | 122 | 1.922 |
| **GUSB** | β-D-glucuronidase | Hs00939627_m1 | 96 | 1.82 |
| **HMBS** | hydroxymethylbilane synthase | Hs00609297_m1 | 64 | 1.965 |
| **HPRT1** | hypoxanthine phosphoribosyltransferase 1 | Hs02800695_m1 | 82 | 1.973 |
| **IPO8** | importin 8 | Hs00183533_m1 | 71 | 1.905 |
| **PGK1** | phosphoglycerate kinase 1 | Hs99999906_m1 | 75 | 1.812 |
| **POLR2A** | RNA polymerase II, subunit A | Hs00172187_m1 | 61 | 1.881 |
| **PPIA** | peptidylprolyl isomerase A (cyclophilin A) | Hs04194521_s1 | 97 | 2.035 |
| **RPLP0** | ribosomal protein lateral stalk subunit P0 | Hs99999902_m1 | 105 | 1.776 |
| **TBP** | TATA box binding protein | Hs00427620_m1 | 91 | 1.81 |
| **TFRC** | transferrin receptor | Hs99999911_m1 | 105 | 1.844 |
| **UBC** | ubiquitin C | Hs00824723_m1 | 71 | 2.057 |
| **YWHAZ** | tyrosine3-monooxygenase/tryptophan  5-monooxygenase activation protein zeta | Hs01122445_g1 | 62 | 1.8 |
| **MYH6** | myosin heavy chain 6 | Hs00411887_m1 | 59 | 1.875 |
| **MYH7** | myosin heavy chain 7 | Hs01110602_m1 | 83 | 1.837 |

**Supplementary Table 2**

RNA integrity numbers of individual samples (RINs > 5 are suitable for qPCR analysis).

| **Sample** | **RIN** |
| --- | --- |
| Control 1 | 8.1 |
| Control 2 | 7.9 |
| Control 3 | 8.3 |
| Control 4 | 7.6 |
| Control 5 | 7.8 |
| HCM 1 | 8.2 |
| HCM 2 | 9.6 |
| HCM 3 | 9.1 |
| HCM 4 | 8.7 |
| ICM 1 | 7.8 |
| ICM 2 | 7.3 |
| ICM 3 | 7 |
| ICM 4 | 8.9 |
| ICM 5 | 8.7 |
| CAD 1 | 7.5 |
| CAD 2 | 8 |
| CAD 3 | 7.5 |
| CAD 4 | 6.9 |
| CAD 5 | 8.7 |
| CAD 6 | 8.8 |
| CAD 7 | 8 |
| CAD 8 | 8 |
| CAD 9 | 7.2 |
| DCM 1 | 7.3 |
| DCM 2 | 7.6 |
| DCM 3 | 8.5 |
| DCM 4 | 7.4 |
| DCM 5 | 8.3 |
| DCM 6 | 8.6 |
| DCM 7 | 6.8 |
| DCM 8 | 8.5 |
| DCM 9 | 8.1 |
| DCM 10 | 8 |

**Supplementary Table 3**

Mean, range, standard deviation (SD) and coefficient of variance (CV) of quantification cycle (Cq) values of individual CRGs.

| **Gene** | **Cq - mean** | **Range** | **Standard deviation** | **Coefficient of variance**  **(%)** |
| --- | --- | --- | --- | --- |
| ACTB | 27.537 | (25.246 – 29.319) | 0.839 | 3.0 |
| B2M | 26.227 | (24.595 – 27.166) | 0.651 | 2.5 |
| GAPDH | 25.080 | (24.158 – 25.837) | 0.407 | 1.6 |
| GUSB | 32.970 | (32.017 – 34.451) | 0.547 | 1.7 |
| HMBS | 31.454 | (29.791 – 33.791) | 0.698 | 2.2 |
| HPRT1 | 31.737 | (30.985 – 32.382) | 0.334 | 1.1 |
| IPO8 | 31.053 | (30.175 – 32.162) | 0.370 | 1.2 |
| PGK1 | 28.107 | (27.518 – 29.085) | 0.381 | 1.4 |
| POLR2A | 30.829 | (30.054 – 31.828) | 0.341 | 1.1 |
| PPIA | 32.736 | (31.453 – 34.553) | 0.599 | 1.8 |
| RPLP0 | 28.045 | (25.951 – 29.152) | 0.753 | 2.7 |
| TBP | 32.308 | (31.733 – 33.244) | 0.312 | 1.0 |
| TFRC | 31.205 | (29.129 – 32.757) | 0.777 | 2.5 |
| UBC | 26.304 | (25.555 – 27.342) | 0.420 | 1.6 |
| YWHAZ | 28.248 | (26.882 – 29.109) | 0.407 | 1.4 |

**Supplementary Table 4**

Mean, range, standard deviation (SD) and coefficient of variance (CV) of quantification cycle Cq values of individual CRGs in all patient groups (HCM – hypertrophic cardiomyopathy, DCM – dilated cardiomyopathy, ICM – ischemic cardiomyopathy, CAD – coronary artery disease).

| **Gene** | **Group** | **Cq - mean** | **Range** | **Standard deviation** | **Coefficient of variance**  **(%)** |
| --- | --- | --- | --- | --- | --- |
| ACTB | Control | 26.162 | (25.246 – 26.885) | 0.687 | 2.6 |
|  | HCM | 27.278 | (26.968 – 27.754) | 0,291 | 1.1 |
|  | ICM | 27.554 | (27.149 – 28.031) | 0.349 | 1.3 |
|  | CAD | 27.882 | (26.580 – 28.611) | 0.650 | 2.3 |
|  | DCM | 28.010 | (27.313 – 29.319) | 0.554 | 2.0 |
| B2M | Control | 25.161 | (24.595 – 25.543) | 0.314 | 1.2 |
|  | HCM | 26.178 | (25.808 – 26.972) | 0.468 | 1.8 |
|  | ICM | 26.393 | (25.968 – 26.646) | 0.231 | 0.9 |
|  | CAD | 26.243 | (24.733 – 26.883) | 0.587 | 2.2 |
|  | DCM | 26.692 | (25.701 – 27.166 | 0.413 | 1.5 |
| GAPDH | Control | 25.332 | (25.119 – 25.837) | 0.259 | 1.0 |
|  | HCM | 24.768 | (24.333 – 25.529) | 0.456 | 1.8 |
|  | ICM | 24.953 | (24.683 – 25.368) | 0.255 | 1.0 |
|  | CAD | 25.089 | (24.715 – 25.669) | 0.370 | 1.5 |
|  | DCM | 25.135 | (24.158 – 25.759) | 0.439 | 1.7 |
| GUSB | Control | 32.537 | (32.017 – 32.990) | 0.369 | 1.1 |
|  | HCM | 32.626 | (32.185 – 33.189) | 0.374 | 1.1 |
|  | ICM | 32.772 | (32.467 – 33.074) | 0.224 | 0.7 |
|  | CAD | 33.156 | (32.319 – 33.854) | 0.544 | 1.6 |
|  | DCM | 33.256 | (32.288 – 34.451 | 0.546 | 1.6 |
| HMBS | Control | 30.931 | (29.791 – 31.674 | 0.677 | 2.2 |
|  | HCM | 31.330 | (31.183 – 31.534 | 0.135 | 0.4 |
|  | ICM | 31.147 | (30.778 – 31.431) | 0.232 | 0.7 |
|  | CAD | 31.597 | (30.759 – 32.199) | 0.437 | 1.4 |
|  | DCM | 31.789 | (30.712 – 33.791) | 0.923 | 2.9 |
| HPRT1 | Control | 31.932 | (31.391 – 32.382) | 0.436 | 1.4 |
|  | HCM | 31.429 | (30.985 – 32.053) | 0.455 | 1.4 |
|  | ICM | 31.629 | (31.170 – 31.952) | 0.256 | 0.8 |
|  | CAD | 31.743 | (31.347 – 32.145) | 0.210 | 0.7 |
|  | DCM | 31.810 | (31.461 – 32.146 | 0.246 | 0.8 |
| IPO8 | Control | 31.083 | (30.757 – 31.361) | 0.245 | 0.8 |
|  | HCM | 30.651 | (30.175 – 31.159) | 0.354 | 1.2 |
|  | ICM | 30.950 | (30.631 – 31.107) | 0.176 | 0.6 |
|  | CAD | 31.038 | (30.764 – 31.321) | 0.152 | 0.5 |
|  | DCM | 31.262 | (30.690 – 32.162) | 0.470 | 1.5 |
| PGK1 | Control | 28.185 | (27.711 – 28.365) | 0.239 | 0.8 |
|  | HCM | 27.880 | (27.518 – 28.409 | 0.326 | 1.2 |
|  | ICM | 27.874 | (27.639 – 28.062) | 0.179 | 0.6 |
|  | CAD | 28.127 | (27.593 – 28.976) | 0.418 | 1.5 |
|  | DCM | 28.257 | (27.593 – 29.085) | 0.402 | 1.4 |
| POLR2A | Control | 31.101 | (30.842 – 31.731) | 0.321 | 1.0 |
|  | HCM | 30.415 | (30.054 – 30.768) | 0.262 | 0.9 |
|  | ICM | 30.757 | (30.394 – 31.020) | 0.236 | 0.8 |
|  | CAD | 30.842 | (30.663 – 31.048) | 0.112 | 0.4 |
|  | DCM | 30.880 | (30.496 – 31.828) | 0.408 | 1.3 |
| PPIA | Control | 32.394 | (31.453 – 33.168) | 0.590 | 1.8 |
|  | HCM | 32.263 | (31.676 – 32.924) | 0.511 | 1.6 |
|  | ICM | 32.522 | (31.733 – 32.908) | 0.408 | 1.3 |
|  | CAD | 32.942 | (32.492 – 33.433) | 0.316 | 1.0 |
|  | DCM | 33.017 | (32.325 – 34.553) | 0.665 | 2.0 |
| RPLP0 | Control | 26.944 | (25.951 – 27.913) | 0.728 | 2.7 |
|  | HCM | 27.665 | (27.123 – 28.229) | 0.382 | 1.4 |
|  | ICM | 27.822 | (27.447 – 28.117) | 0.248 | 0.09 |
|  | CAD | 28.323 | (27.766 – 29.140) | 0.419 | 1.5 |
|  | DCM | 28.610 | (27.495 – 29.152) | 0.530 | 1.9 |
| TBP | Control | 32.640 | (32.384 – 32.927) | 0.182 | 0.6 |
|  | HCM | 32.267 | (32.157 – 32.413) | 0.103 | 0.3 |
|  | ICM | 32.207 | (31.894 – 32.408) | 0.173 | 0.5 |
|  | CAD | 32.160 | (31.798 – 32.517) | 0.230 | 0.7 |
|  | DCM | 32.341 | (31.733 – 33.244) | 0.395 | 1.2 |
| TFRC | Control | 30.740 | (29.129 – 32.024) | 1.079 | 3.5 |
|  | HCM | 30.551 | (30.166 – 31.264) | 0.423 | 1.4 |
|  | ICM | 31.624 | (30.836 – 32.257) | 0.539 | 1.7 |
|  | CAD | 31.219 | (29.967 – 31.843) | 0.597 | 1.9 |
|  | DCM | 31.476 | (30.495 – 32.757) | 0.657 | 2.1 |
| UBC | Control | 26.565 | (25.998 – 27.020) | 0.403 | 1.5 |
|  | HCM | 26.002 | (25.555 – 26.468) | 0.338 | 1.3 |
|  | ICM | 26.092 | (25.829 – 26.542) | 0.247 | 0.9 |
|  | CAD | 26.158 | (25.862 – 26.648) | 0.230 | 0.9 |
|  | DCM | 26.532 | (25.923 – 27.342) | 0.463 | 1.7 |
| YWHAZ | Control | 27.695 | (26.882 – 28.013) | 0.429 | 1.5 |
|  | HCM | 28.067 | (27.802 – 28.282) | 0.172 | 0.6 |
|  | ICM | 28.101 | (27.933 – 28.320) | 0.159 | 0.6 |
|  | CAD | 28.561 | (28.070 – 28.914) | 0.266 | 0.9 |
|  | DCM | 28.388 | (28.056 – 29.109) | 0.286 | 1.0 |

**Supplementary Table 5**

Cq values of individual genes in each sample.

| **Sample** | **POLR2A** | **GAPDH** | **B2M** | **ACTB** | **HPRT1** | **GUSB** | **HMBS** | **IPO8** | **PGK1** | **PPIA** | **RPLP0** | **TBP** | **TRFC** | **UBC** | **YWHAZ** |
| --- | --- | --- | --- | --- | --- | --- | --- | --- | --- | --- | --- | --- | --- | --- | --- |
| Contr. 1 | 30.955 | 25.177 | 25.543 | 26.885 | 32.338 | 32.783 | 31.439 | 31.361 | 28.258 | 33.168 | 27.301 | 32.927 | 31.303 | 26.869 | 28.013 |
| Contr. 2 | 30.941 | 25.119 | 24.595 | 25.418 | 31.391 | 32.017 | 30.580 | 30.757 | 27.711 | 32.149 | 25.951 | 32.384 | 29.837 | 26.174 | 26.882 |
| Contr. 3 | 30.842 | 25.243 | 25.320 | 25.246 | 31.427 | 32.194 | 29.791 | 30.829 | 28.365 | 31.453 | 26.257 | 32.662 | 29.129 | 25.998 | 27.636 |
| Contr. 4 | 31.047 | 25.286 | 25.208 | 26.674 | 32.382 | 32.702 | 31.171 | 31.293 | 28.286 | 32.363 | 27.301 | 32.522 | 31.407 | 26.763 | 27.962 |
| Contr. 5 | 31.731 | 25.837 | 25.141 | 26.587 | 32.122 | 32.990 | 31.674 | 31.175 | 28.303 | 32.836 | 27.913 | 32.705 | 32.024 | 27.020 | 27.980 |
| HCM 1 | 30.519 | 24.673 | 25.869 | 27.167 | 31.675 | 32.420 | 31.183 | 30.541 | 27.769 | 31.867 | 27.528 | 32.312 | 30.339 | 25.851 | 28.072 |
| HCM 2 | 30.319 | 24.536 | 26.061 | 27.222 | 30.985 | 32.709 | 31.534 | 30.731 | 28.409 | 32.587 | 27.730 | 32.186 | 30.434 | 26.136 | 28.282 |
| HCM 3 | 30.054 | 24.333 | 25.808 | 26.968 | 31.004 | 32.185 | 31.237 | 30.175 | 27.518 | 31.676 | 27.173 | 32.157 | 30.166 | 25.555 | 27.802 |
| HCM 4 | 30.768 | 25.529 | 26.972 | 27.754 | 32.053 | 33.189 | 31.368 | 31.159 | 27.825 | 32.924 | 28.229 | 32.413 | 31.264 | 26.468 | 28.113 |
| ICM 1 | 31.020 | 25.368 | 26.646 | 27.572 | 31.952 | 32.984 | 31.345 | 30.887 | 28.016 | 32.604 | 27.644 | 32.206 | 31.897 | 26.093 | 27.933 |
| ICM 2 | 30.394 | 24.878 | 25.968 | 27.184 | 31.170 | 32.467 | 31.034 | 30.631 | 27.639 | 31.733 | 27.447 | 32.408 | 31.187 | 25.829 | 27.938 |
| ICM 3 | 30.848 | 24.683 | 26.374 | 27.149 | 31.634 | 32.671 | 31.149 | 31.057 | 28.062 | 32.717 | 28.117 | 32.214 | 32.257 | 25.911 | 28.249 |
| ICM 4 | 30.947 | 25.109 | 26.530 | 28.031 | 31.736 | 33.074 | 31.431 | 31.068 | 27.977 | 32.908 | 27.866 | 32.314 | 31.970 | 26.087 | 28.320 |
| ICM 5 | 30.578 | 24.727 | 26.447 | 27.836 | 31.653 | 32.663 | 30.778 | 31.107 | 27.677 | 32.650 | 28.035 | 31.894 | 30.806 | 26.542 | 28.063 |
| ICM 1 | 30.807 | 24.752 | 26.120 | 27.299 | 31.347 | 32.435 | 30.759 | 31.011 | 27.775 | 32.492 | 27.766 | 32.192 | 31.195 | 26.318 | 28.070 |
| ICM 2 | 30.905 | 25.441 | 24.733 | 26.580 | 31.612 | 32.319 | 32.199 | 31.321 | 28.512 | 33.211 | 28.740 | 31.905 | 30.377 | 26.192 | 28.579 |
| CAD 1 | 30.663 | 24.985 | 26.002 | 27.367 | 31.788 | 32.812 | 31.681 | 31.053 | 27.593 | 33.101 | 27.970 | 31.798 | 31.843 | 25.862 | 28.389 |
| CAD 2 | 30.775 | 24.752 | 26.529 | 28.097 | 31.728 | 32.986 | 31.535 | 30.764 | 28.198 | 32.631 | 28.075 | 32.234 | 29.967 | 25.906 | 28.763 |
| CAD 3 | 30.774 | 24.874 | 26.306 | 28.017 | 32.145 | 33.078 | 31.861 | 30.963 | 28.356 | 33.313 | 28.518 | 32.502 | 31.664 | 26.314 | 28.830 |
| CAD 4 | 30.989 | 25.662 | 26.883 | 28.611 | 31.847 | 33.777 | 31.430 | 31.096 | 27.991 | 32.768 | 28.001 | 32.517 | 31.564 | 26.025 | 28.781 |
| CAD 5 | 31.048 | 25.669 | 26.624 | 28.523 | 31.834 | 33.854 | 32.153 | 31.205 | 28.976 | 33.433 | 29.140 | 32.193 | 31.632 | 26.648 | 28.914 |
| CAD 6 | 30.807 | 24.715 | 26.542 | 27.859 | 31.558 | 33.392 | 31.115 | 31.008 | 27.665 | 32.639 | 28.130 | 31.995 | 31.453 | 26.107 | 28.356 |
| CAD 7 | 30.809 | 24.951 | 26.367 | 28.590 | 31.824 | 33.753 | 31.641 | 30.925 | 28.079 | 32.889 | 28.564 | 32.104 | 31.274 | 26.049 | 28.370 |
| CAD 8 | 31.058 | 25.540 | 25.701 | 27.726 | 32.043 | 32.971 | 32.152 | 31.775 | 28.801 | 32.696 | 28.802 | 32.163 | 31.976 | 27.342 | 28.245 |
| CAD 9 | 30.506 | 25.016 | 26.711 | 27.313 | 31.964 | 32.984 | 31.002 | 30.844 | 28.245 | 32.466 | 28.582 | 32.313 | 31.435 | 26.048 | 28.276 |
| DCM 1 | 30.604 | 24.843 | 26.725 | 28.468 | 31.463 | 33.671 | 31.677 | 31.141 | 28.261 | 32.966 | 28.928 | 32.293 | 31.227 | 26.211 | 28.056 |
| DCM 2 | 30.548 | 25.467 | 27.107 | 27.852 | 32.146 | 32.978 | 30.839 | 30.690 | 27.995 | 33.000 | 28.905 | 32.073 | 31.424 | 26.561 | 28.456 |
| DCM 3 | 30.588 | 24.945 | 26.405 | 27.749 | 31.787 | 33.188 | 30.712 | 30.865 | 27.593 | 32.733 | 28.309 | 32.287 | 31.166 | 25.993 | 28.182 |
| DCM 4 | 30.860 | 24.929 | 26.802 | 28.146 | 32.090 | 32.991 | 32.082 | 31.531 | 28.025 | 32.678 | 29.152 | 32.043 | 31.785 | 26.916 | 28.222 |
| DCM 5 | 31.060 | 25.203 | 26.594 | 28.270 | 31.552 | 33.523 | 31.417 | 30.992 | 28.280 | 32.778 | 29.000 | 32.560 | 31.985 | 26.475 | 28.441 |
| DCM 6 | 30.496 | 24.158 | 26.562 | 27.399 | 31.461 | 32.288 | 31.317 | 30.914 | 27.997 | 32.325 | 27.495 | 31.733 | 30.506 | 25.923 | 28.261 |
| DCM 7 | 31.255 | 25.759 | 27.166 | 27.856 | 31.908 | 33.513 | 32.905 | 31.709 | 28.288 | 33.980 | 27.848 | 32.698 | 30.495 | 26.811 | 28.631 |
| DCM 8 | 31.828 | 25.493 | 27.144 | 29.319 | 31.687 | 34.451 | 33.791 | 32.162 | 29.085 | 34.553 | 29.080 | 33.244 | 32.757 | 27.042 | 29.109 |
| DCM 9 | 30.955 | 25.177 | 25.543 | 26.885 | 32.338 | 32.783 | 31.439 | 31.361 | 28.258 | 33.168 | 27.301 | 32.927 | 31.303 | 26.869 | 28.013 |
| DCM 10 | 30.941 | 25.119 | 24.595 | 25.418 | 31.391 | 32.017 | 30.580 | 30.757 | 27.711 | 32.149 | 25.951 | 32.384 | 29.837 | 26.174 | 26.882 |

**Supplementary Table 6**

Stability values of CRGs in LV of failing human hearts (after the elimination of variantly expressed genes) based on geNorm, NormFinder, BestKeeper and Delta Cq analysis. The most stable genes are indicated by the lowest M-value (geNorm), stability value (NormFinder) and mean SD (Delta Cq) as well as the highest correlation coefficient to BestKeeper index (BestKeeper).

| **Gene** | **geNorm**  (M-value) | **NormFinder**  (stability value) | **BestKeeper**  (correlation coefficient to BestKeeper index) | **Delta Cq**  (mean SD) |
| --- | --- | --- | --- | --- |
| GAPDH | 0.280 | 0.187 | 0.701 | 0.47 |
| GUSB | 0.392 | 0.178 | 0.763 | 0.519 |
| HMBS | 0.431 | 0.258 | 0.799 | 0.596 |
| HPRT1 | 0.327 | 0.161 | 0.594 | 0.481 |
| IPO8 | 0.273 | 0.095 | 0.866 | 0.402 |
| PGK1 | 0.311 | 0.146 | 0.697 | 0.457 |
| POLR2A | 0.256 | 0.195 | 0.805 | 0.418 |
| PPIA | 0.363 | 0.179 | 0.862 | 0.5 |
| TFRC | 0.469 | 0.253 | 0.707 | 0.706 |
| UBC | 0.298 | 0.171 | 0.781 | 0.446 |

**Supplementary Table 7**

Stability values and significance levels of CRGs in random groups of 5 samples (5 diseased groups and a control group). Since all groups are of equal size (5 samples each, including the control group), F-ANOVA test was used. In bold, p ˂ 0.05.

| Gene | Variance component | | F | Significance | V_B_ | V_W_ | Stability index | Ranking |
| --- | --- | --- | --- | --- | --- | --- | --- | --- |
|  | Between group | Within group |  |  |  |  |  |  |
| IPO8 | 0.029 | 0.067 | 0.435 | 0.820 | 0.052 | 0.120 | 0.006 | 1 |
| **YWHAZ** | 0.155 | 0.046 | 3.405 | **0.018** | 0.194 | 0.057 | 0.011 | **2** |
| HPRT1 | 0.057 | 0.058 | 0.982 | 0.449 | 0.112 | 0.114 | 0.013 | 3 |
| POLR2A | 0.074 | 0.065 | 1.113 | 0.371 | 0.135 | 0.119 | 0.016 | 4 |
| TBP | 0.060 | 0.036 | 1.671 | 0.180 | 0.169 | 0.101 | 0.017 | 5 |
| GAPDH | 0.086 | 0.063 | 1.373 | 0.270 | 0.155 | 0.113 | 0.018 | 6 |
| PGK1 | 0.061 | 0.053 | 1.136 | 0.368 | 0.169 | 0.148 | 0.025 | 7 |
| TFRC | 0.173 | 0.241 | 0.716 | 0.618 | 0.143 | 0.200 | 0.029 | 8 |
| **RPLP0** | 0.468 | 0.127 | 3.699 | **0.013** | 0.404 | 0.109 | 0.044 | **9** |
| UBC | 0.150 | 0.084 | 1.784 | 0.154 | 0.284 | 0.159 | 0.045 | 10 |
| PPIA | 0.197 | 0.156 | 1.266 | 0.310 | 0.243 | 0.192 | 0.047 | 11 |
| HMBS | 0.303 | 0.225 | 1.347 | 0.279 | 0.269 | 0.200 | 0.054 | 12 |
| GUSB | 0.154 | 0.137 | 1.122 | 0.375 | 0.253 | 0.226 | 0.057 | 13 |
| **ACTB** | 0.783 | 0.109 | 7.215 | **0.0003** | 0.645 | 0.089 | 0.058 | **14** |
| **B2M** | 0.572 | 0.096 | 5.970 | **0.001** | 0.588 | 0.099 | 0.058 | **15** |

**Supplementary Table 8**

Stability values and significance levels of CRGs in individual patient groups. Since the patient groups are of non-equal size, p-values were calculated using Welch’s ANOVA test. In bold, p ˂ 0.05.

| Gene | Variance component | | F | Significance | V_B_ | V_W_ | Stability index | Ranking |
| --- | --- | --- | --- | --- | --- | --- | --- | --- |
|  | Between group | Within group |  |  |  |  |  |  |
| **YWHAZ** | 0.246 | 0.032 | 7.623 | **0.009** | 0.306 | 0.040 | 0.012 | **1** |
| GAPDH | 0.074 | 0.060 | 1.235 | 0.323 | 0.133 | 0.108 | 0.014 | 2 |
| HPRT1 | 0.079 | 0.051 | 1.535 | 0.577 | 0.154 | 0.100 | 0.015 | 3 |
| IPO8 | 0.119 | 0.050 | 2.366 | 0.305 | 0.210 | 0.089 | 0.019 | 4 |
| **TBP** | 0.076 | 0.032 | 2.385 | **0.027** | 0.218 | 0.091 | 0.020 | **5** |
| POLR2A | 0.141 | 0.051 | 2.749 | 0.136 | 0.26 | 0.09 | 0.024 | 6 |
| PGK1 | 0.065 | 0.051 | 1.269 | 0.191 | 0.186 | 0.146 | 0.027 | **7** |
| TFRC | 0.388 | 0.195 | 1.987 | 0.102 | 0.315 | 0.158 | 0.050 | 8 |
| PPIA | 0.287 | 0.128 | 2.253 | 0.167 | 0.354 | 0.157 | 0.056 | 9 |
| UBC | 0.213 | 0.078 | 2.738 | 0.155 | 0.395 | 0.144 | 0.057 | 10 |
| **RPLP0** | 0.888 | 0.093 | 9.513 | **0.007** | 0.738 | 0.078 | 0.057 | **11** |
| HMBS | 0.366 | 0.210 | 1.743 | 0.163 | 0.326 | 0.187 | 0.061 | 12 |
| **B2M** | 0.741 | 0.085 | 8.681 | **0.0003** | 0.745 | 0.086 | 0.064 | **13** |
| **ACTB** | 0.957 | 0.107 | 8.961 | **0.008** | 0.771 | 0.086 | 0.066 | **14** |
| GUSB | 0.292 | 0.109 | 2.685 | 0.096 | 0.469 | 0.175 | 0.082 | 15 |

**Supplementary Table 9**

Expression of MYH6 and MYH7 genes in control and failing human left ventricle samples after normalization with IPO8 and POLR2A. P-values were calculating using Mann-Whitney nonparametric test, p-values ˂ 0.05 are in bold, power of the test – 100% for both genes.

| Gene | p-value | Comparison | Ratio | 95% Cl low | 95% Cl high |
| --- | --- | --- | --- | --- | --- |
| MYH6 | **0.00013** | control/failing | 7.509 | 3.442 | 16.380 |
| MYH7 | **˂0.0001** | control/failing | 0.228 | 0.161 | 0.323 |

**Supplementary Table 10**

Comparison of MYH6 gene expression between three datasets – this study and two different RNAseq datasets (Molina-Navarro et al., 2014 – reference [31]; Sweet et al., 2018 – reference [32]).

|  | Fold change | | | | | |
| --- | --- | --- | --- | --- | --- | --- |
|  | This study (qPCR) | | RNAseq GSE55296 | | RNAseq GSE116250 | |
|  | ICM | DCM | ICM | DCM | ICM | DCM |
| MYH6 | **-5.50** | **-8.52** | **-2.65** | **-2.87** | **-1.61** | **-1.54** |

**Supplementary Table 11**

Expression of ACTB and B2M genes in control and failing human left ventricle samples after normalization with IPO8 and POLR2A. P-values were calculating using Mann-Whitney nonparametric test, p-values ˂ 0.05 are in bold, power of the test – 100% for both genes.

| Gene | p-value | Comparison | Ratio | 95% Cl low | 95% Cl high |
| --- | --- | --- | --- | --- | --- |
| ACTB | **˂0.0001** | control/failing | 2.734 | 2.088 | 3.579 |
| B2M | **0.0004** | control/failing | 2.443 | 1.781 | 3.352 |
